# Type 2β Corticotrophin Releasing Factor Receptor Forms a Heteromeric Complex With Dopamine D1 Receptor in Living Cells

**DOI:** 10.3389/fphar.2019.01501

**Published:** 2020-01-08

**Authors:** Hector E. Yarur, Maria Estela Andrés, Katia Gysling

**Affiliations:** Department of Cellular and Molecular Biology, Faculty of Biological Sciences, Pontificia Universidad Católica de Chile, Santiago, Chile

**Keywords:** D1R (dopamine D1 receptor), heteromer, GPCR (G protein-coupled receptors, A7r5 cells, CRF2β

## Abstract

Corticotrophin releasing factor (CRF) and its related peptides differentially bind to CRF receptors to modulate stress-related behaviors. CRF receptors comprise two G-protein coupled receptors (GPCR), type-1 CRF receptors (CRF1), and type-2 CRF receptors (CRF2). CRF2 encompasses three spliced variants in humans, alpha (CRF2α), beta (CRF2β), and gamma (CRF2γ), which differ in their N-terminal extracellular domains and expression patterns. Previously, we showed that CRF2α form a heteromeric protein complex with dopamine D1 receptors (D1R), leading to changes in the signaling of D1R. Based on the high sequence identity between CRF2α and CRF2β, we hypothesized that CRF2β also heteromerize with D1R. To test the hypothesis, we compared the expression and localization of both CRF2 isoforms and whether CRF2β form stable protein complexes with D1R in HEK293 and ATR75 cell lines. We observed that the immunoreactivity for CRF2β was similar to that of CRF2α in the endoplasmic compartment but significantly higher in the Golgi compartment. Immunoprecipitation analysis showed that CRF2β forms a heteromeric protein complex with D1R. Furthermore, the protein complex formed by CRF2β and D1R was stable enough to change the sub-cellular localization of CRF2β when it was co-expressed with a construct of D1R bearing a nuclear localization signal. Immunofluorescence in A7R5 cells, which endogenously express CRF2β and D1R, shows significant colocalization of CRF2β with D1R. In conclusion, our results show that CRF2β forms a stable heteromeric protein complex with D1R, a potential new therapeutic target in tissues where both receptors are co-expressed, such as the septum in the brain, and heart, kidney, and skeletal muscle in the periphery.

## Introduction

Corticotrophin releasing factor (CRF) constitutes a key component in the animal stress response (Vale et al., 1981; Snyder et al., 2015). The CRF system has three additional peptides, urocortin (UCN) 1, UCN2, and UCN3 (Hillhouse and Grammatopoulos, 2006). CRF peptides signal through two G-protein coupled receptors (GPCR), type-1 (CRF1) and type-2 (CRF2) receptors (Dautzenberg and Hauger, 2002). CRF1 and CRF2 receptors have high amino acid sequence identity but a distinct affinity for CRF peptides (Chalmers et al., 1996). CRF has a higher affinity for CRF1 than for CRF2, (Rutz et al., 2006; Deussing and Chen, 2018). UCN1 has higher affinity than CRF for CRF1 and CRF2, while UCN2 and UCN3 are highly selective for CRF2 (De Souza, 1995).

Three splice variants of CRF2 (α, β, and γ) are expressed in several human tissues (Lovenberg et al., 1995a; Kostich et al., 1998). In rodents and humans, the CRF2α splice variant presents a high density in the central nervous system, and the CRF2β splice variant is primarily expressed in peripheral tissue (Lovenberg et al., 1995b; Perrin et al., 1995). In humans, CRF2β is present in some brain regions and peripheral tissues (Kostich et al., 1998). CRF2 splice variants differ only in their N-terminal extracellular domains, which confer significant differences in their sub-cellular localization (Schulz et al., 2010; Teichmann et al., 2012; Slater et al., 2016a). It has been described that CRF and dopamine (DA) signaling contributes to responses, such as stress-related response (Orozco-Cabal et al., 2008). Thus, the understanding of the mechanisms by which DA and CRF interact could lead to reveal the mechanisms by which these neurotransmitters modulate the behavioral response.

We and other groups have shown that CRF receptors form heteromers with other GPCRs (Fuenzalida et al., 2014; Navarro et al., 2015; Hasdemir et al., 2017). Particularly, the CRF2β isoform assembles into heteromeric complexes with CRF1 (Hasdemir et al., 2017) and the CRF2α isoform assembles with the dopamine D1 receptor (D1R, Fuenzalida et al., 2014). CRF2α/D1R heteromer displays distinct signaling properties than D1R and CRF2α alone (Fuenzalida et al., 2014).

Since CRF2α and CRF2β share a high protein sequence similarity and CRF2α can interact with D1R, we decided to study whether CRF2β forms also a stable heteromeric complex with D1R. To this end, we first compared the sub-cellular localization of recombinant CRF2α and CRF2β expressed in HEK293 cells. Next, we studied if CRF2β can form a stable protein complex with D1R. To this end we used the strategy designed by O’Dowd et al. (2005) consisting in: a) the addition of a nuclear localization signal (nls) to D1R (D1Rnls) that translocate the receptor and its eventual partner CRf2o the nucleus and b) the use of butaclamol, a reverse agonist of D1R that retains D1Rnls and its partner in the plasmatic membrane. We also evaluated the degree of colocalization between D1R and CRF2β in the A7R5 cell line, derived from vascular smooth muscle, that has been shown to express D1R (Chen et al., 2018) and CRF2β (Kageyama et al., 2000; Hoare et al., 2005). Our results show, that CRF2β can form a stable protein complex with D1R and that both receptors are significantly colocalized in A7R5 cells.

## Material And Methods

### Cell Culture and Transfection

HEK293T cells and A7R5 cells (kindly donated by Dr. Mario Chiong, University of Chile) were grown with DMEM (Gibco) supplemented with 10% FBS (HyClone Labs), 1% penicillin/streptomycin 100× (Gibco), and 2 mM GlutaMax (Gibco). Plasmids were transfected using Lipofectamine 2000 (Invitrogen) according to the manufacturer’s instructions and as previously described (Fuenzalida et al., 2014). Experiments were performed 48 h after plasmids transfection. The ratio of transfected plasmid was 1:1 and the amount of DNA transfected for immunofluorescence was 500 ng of total DNA and for immunoprecipitation experiments was 8 ug of total DNA.

### Expression Vectors

pcDNA3.1/Myc-His/D1R, pcDNA3.1/myc-His/D1Rnls, and pcDNA3.1/HA-CRF2α were previously described (Fuenzalida et al., 2014; Slater et al., 2016b). The pcDNA3.1/HA-CRF2β was commercially obtained (GeneCopoeia). The HA epitope in both receptors is located in their N terminal.

### Protein Extraction and Immunoprecipitation

After treatments, HEK293T cells were collected in ice-cold PBS (pH 7.4), centrifuged at 1500 rpm for 5 min and resuspended in lysis buffer (50 mM Tris-HCl, pH 8.0, 150 mM NaCl, 1 mM EDTA, 0.1% SDS, and 1% Triton X-100) with the Protease Inhibitor Cocktail cOmplete Mini (Roche) as described by Rutz et al. (2006). Cells were then homogenized with three pulses of 10 s using a piston sonicator (Cell Ultrasonic Disrupter) and incubated for at least 1 h at 4°C. Finally, the homogenate was centrifuged at 15,000 rpm for 15 min at 4°C. The supernatant was collected, and protein concentration determined with the Micro BCA Protein Assay Kit (Thermo Scientific). Co-immunoprecipitation assays were performed essentially as previously described (Fuenzalida et al., 2014). Seven hundred µg of protein extract were pre-cleared with TrueBlot^®^ anti-Rabbit Ig IP Agarose Beads (Rockland) and incubated with 1 µg of rabbit anti-Myc antibody (Ab9106, Abcam). Loading buffer 2× (8 M urea, 2% SDS, 100 mM DTT, 375 mM Tris, pH 6.8) was added to each sample and heated to 37°C for 1 h to perform western blotting.

### Western Blotting

Proteins were fractionated in 10% sodium dodecyl sulfate–polyacrylamide gel electrophoresis (SDS–PAGE) and then transferred into PVDF membrane (Millipore). Membranes were incubated overnight at 4°C with mouse anti-HA (1:1000, #901501, BioLegend) followed by the peroxidase-conjugated anti-mouse antibody for 2 h (1/4000, Jackson ImmunoResearch Laboratories). The membranes were revealed using SuperSignal West Pico Chemiluminescent Substrate (Pierce Biotechnology).

### Heteromer Mobilization Assay

HEK293T cells were seeded at a density of 7 × 10^6^ cells per well on a 24-well plate on coverslips coated with poly-L-lysine (Sigma). Six hours post-transfection, the cells were treated with 1 μM (+)-butaclamol (D1R antagonist) in supplemented DMEM medium for 48 h (O’Dowd et al., 2005). After washing with PBS, cells were fixed with 4% paraformaldehyde (PFA) and receptors localization analyzed by immunofluorescence.

### Immunofluorescence and Confocal Microscopy

Immunofluorescence assays were done as previously described (Fuenzalida et al., 2014). Cells were incubated with primary antibodies: rabbit anti-D1R (1:500; sc-14001, Santa Cruz Biotechnology), goat anti-CRF2 (1:500; sc-20550, Santa Cruz Biotechnology), mouse anti-HA (1:1000; HA.11 Clone 16B12, Covance Inc), rat anti-KDEL (1:500; ab50601, Abcam); rabbit anti-D1R receptor (1:500; ab20066, Abcam), and rabbit anti-Giantin (1:500; ab80864, Abcam) overnight at 4°C in a wet-chamber. Cells were then washed and incubated for 2 h with the following secondary antibodies: donkey anti-rabbit AlexaFluor^488^, donkey anti-rabbit AlexaFluor^Cy3^, donkey anti-goat AlexaFluor^488^, donkey anti-goat AlexaFluor^Cy3^, and donkey anti-rat AlexaFluor^647^ at room temperature. Cells were washed and mounted with mounting media (Dako).

Images were obtained with a confocal microscope (Fluoview 1000, Olympus) And Fluoview V6.0 software. images were digitally obtained with a 100× objective (N.A. 1.4 Oil). The stacking of images was done with A Z step of 200 Nm per cell. Images were processed using the imagej software (Rsb.Info.Nih.Gov/Ij). The deconvolution analysis was performed using the “Iterative Deconvolve 3D” plugin within imagej as previously described (Blanco et al., 2011) and Manders coefficient And Van Steensel analysis was used for measure colocalization (Manders et al., 1993; Van Steensel et al., 1996). Fluorescence Intensity was used to compare the cellular distribution of the marks.

### Statistical Analysis

Statistical analysis was performed with the GraphPad Prism 6 software (GraphPad Software). The data are expressed as the mean ± SEM. Statistical significance was assessed with unpaired Mann-Whitney *U* test.

## Results

### Subcellular Localization of CRF2 Isoforms Expressed in HEK293 Cells

**Figure 1:**
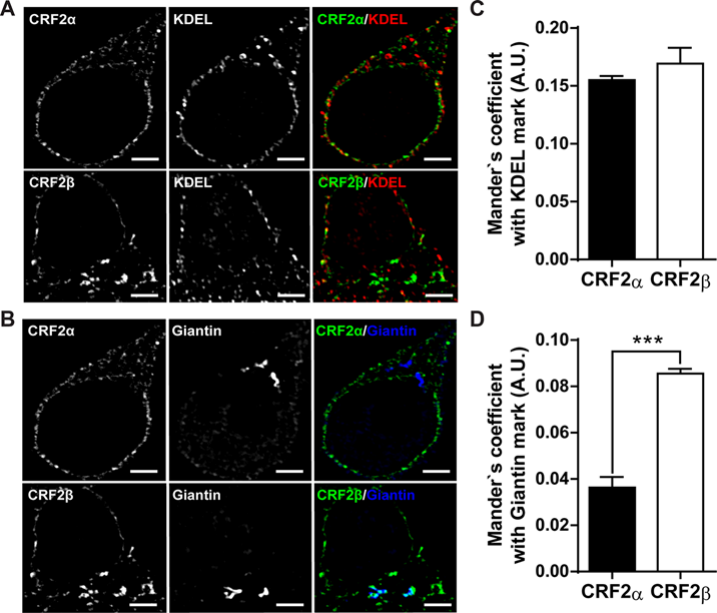
Comparison of the subcellular distribution of CRF2 isoforms in HEK293 cells. **(A** and **B**) Confocal immunodetection of the CRF2 isoforms in a preparation of HEK293 cells (one-plane microphotographs). **(A)** Confocal immunofluorescence for CRF2α or CRF2β (green), using a mouse anti-HA antibody and KDEL (red) (scale bar: 2 μm). **(B)** Confocal immunofluorescence for CRF2α or CRF2β (green) and Giantin (blue) (scale bar: 2 μm). **(C)** Mander’s analyses for co-localization in A. **(D)** Mander’s analyses for co-localization in B. Unpaired Mann-Whitney *U* test compared between CRF2 isoforms (***p < 0.0005). Values are expressed as mean ± SEM, N = 3 and each N represent 7 independent cells analyzed.

The residence time of GPCR in each compartment of the secretory path varies according to their protein sequence that determines specific protein-protein interactions (Chuang and Sung, 1998). To determine the localization of each CRF receptor, we used specific markers for each secretory compartment, KDEL for the endoplasmic reticular compartment, and Giantin for the Golgi compartment (**Figure 1**). As can be seen in **Figure 1**, CRF2α is mostly associated with the KDEL compartment (**Figures 1 A**, **B**), as previously shown (Fuenzalida et al., 2014). The presence of CRF2β in the KDEL compartment was similar to CRF2α (**Figures 1A**, **C**). In contrast, the presence of CRF2β in the Giantin compartment was significantly higher than that of CRF2α (**Figures 1B**, **D**). Overall, these results indicate that the presence of CRF2β in the secretory pathway is significantly higher than CRF2α.

### CRF2β Forms a Protein Complex With D1R

**Figure 2:**
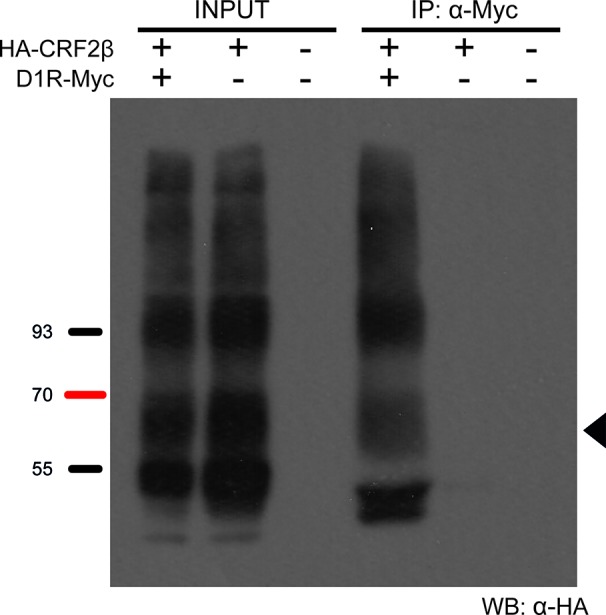
D1R and CRF2β form a protein complex in HEK293 cells. Representative western blot of the co-immunoprecipitation of D1R and CRF2β from HEK293 cells. The protein extract from HEK293 cells expressing CRF2β plus D1R, CRF2β, or empty vector pcDNA were incubated with a rabbit anti-myc antibody for the immunoprecipitation and with a mouse anti-HA antibody for the immunoreactivity for CRF2β. The black arrow shows the estimated molecular weight for CRF2β (~70 kDa). The image was a representation of three replicated experiments. Input line is 5% of the whole protein extraction and IP line is the immunoprecipitation of the protein of interest from the whole protein extraction.

To determine if CRF2β form a protein complex with D1R, we performed co-immunoprecipitation experiments using whole extracts obtained from HEK293 cells transfected with plasmids bearing human HA-CRF2β and Myc-D1R. HA-CRF2β (band of ∼70 kDa) precipitated in the same immunocomplex with Myc-D1R in protein extracts from cells transiently transfected with both receptors (**Figure 2**). The specificity of this interaction is shown by control experiments in which immunoreactivity is not observed when the immunoprecipitations were performed with protein extracts from cells transfected with HA-CRF2β alone or with the empty vectors.

**Figure 3:**
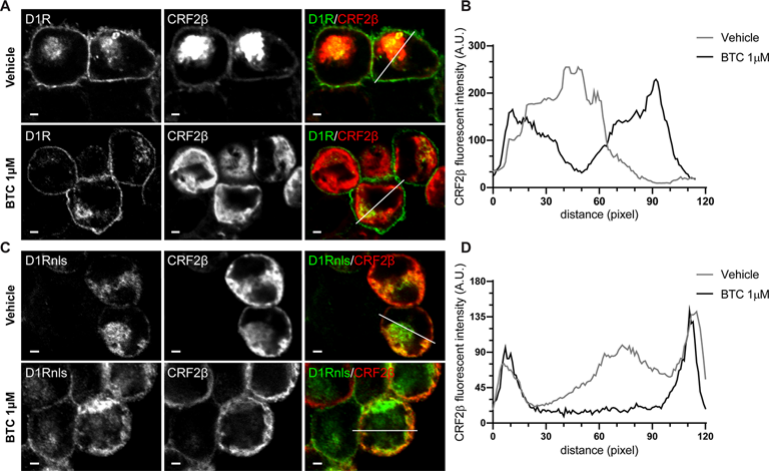
The protein complex of D1R and CRF2β is stable to change their cellular localization. **(A** and **C)** Confocal immunodetection of the CRF2β and D1R or D1Rnls in the presence of 1 μM of Butaclamol (BTC) in HEK293 cells. **(A)** Confocal immunofluorescence for CRF2β (red), using a mouse anti-HA antibody and D1R (green) in the presence of BTC or vehicle (scale bar: 2 μm). **(C)** Confocal immunofluorescence for CRF2β (red) and D1Rnls (green) in the presence of BTC or vehicle (scale bar: 2 μm). **(B)** Quantification of the distribution of CRF2β fluorescence in A. **(D)** Quantification of the distribution of CRF2β fluorescence in C. White lines depict the zone where CRF2β fluorescence was quantified.

To evaluate the stability of the protein complex formed between CRF2β and D1R, we used the heteromer mobilization strategy described by O’Dowd et al. (2005). Through the use of immunofluorescence, we observed that CRF2β and D1R co-localize in intracellular compartments (**Figure 3**). Interestingly, the incubation with 1 μM butaclamol (BTC), specific D1R antagonist, changed the subcellular distribution of CRF2β from a central localization to an out of the center localization in HEK293 cells (**Figures 3A**, **B**) supporting the formation of a stable protein complex between the receptors.

To further test the interaction and stability of the complex between D1R and CRF2β, we studied the localization of CRF2β when it is co-expressed with a mutant recombinant D1R that bears a nls (**Figures 3C**, **D**). We detected a more pronounced co-localization of the CRF2β with D1Rnls (**Figure 3**). The formation of a stable protein complex was further proven using HEK293 cells co-expressing D1Rnls and CRF2β in the presence of BTC. The presence of BTC modified the cellular distribution of CRF2β, from a central location to a peripheral localization (**Figures 3C**, **D**). Taken together, the data show that CRF2β forms a stable protein complex with D1R in HEK293 cells.

### *In Vivo* Visualization of the CRF2β and D1R Heteromer Complex in A7R5 Cells

**Figure 4:**
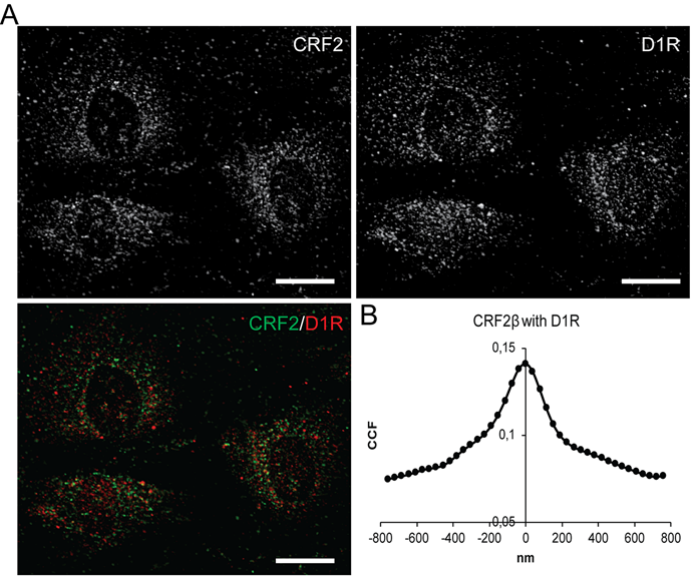
CRF2β and D1R are endogenously express in A7R5 cells and present significant colocalization. **(A)** Confocal immunodetection of CRF2β and D1R in A7R5 cells. **(A)** Confocal immunofluorescence for CRF2β (green) and D1R (red) (scale bar: 20 μm). **(B)** Quantification of the colocalization of CRF2β with D1R by Van Steensel analysis.

The previous data showing that recombinant CRF2β and D1R form a stable protein complex, prompted us to test whether endogenous proteins heteromerize. To this end, we used A7R5 cells derived from vascular smooth muscle cells from rat thoracic aorta that express both receptors (Hoare et al., 2005; Chen et al., 2018). Using immunofluorescence, we corroborated the expression of both CRF2β and D1R in A7R5 cells, which colocalize in these cells (**Figure 4A**). To quantify the extent of colocalization, we applied the method of Van Steensel et al. (1996). As can be seen in **Figure 4B**, the quantitative analysis of CRF2β and D1R labels yielded a “bell shape” curve indicating colocalization, with a colocalization index of 0.140 ± 0.006 (**Figure 4B**).

## Discussion

In the present study, we show that CRF2β differs in its subcellular localization pattern with CRF2α, but also form a stable protein complex with D1R. CRF2α and CRF2β were found mainly in the endoplasmic reticulum; however, CRF2β was associated with the Golgi apparatus in a significantly higher amount than CRF2α. Interestingly, equal to CRF2α, CRF2β can form a stable protein complex with D1R.

CRF2β was found mainly associated with the endoplasmic reticulum; similar to what it has been previously reported for CRF2α (Slater et al., 2016b). However, CRF2β displays a higher association than CRF2α to the Golgi apparatus. Previously, it was reported that overexpressed CRF2β localizes in the plasma membrane of HEK293 cells (Markovic et al., 2008). Our immunofluorescent assays with overexpressed CRF2α and CRF2β in HEK293 cells unequivocally show that the labeling is mainly associated with the endoplasmic reticulum for both CRF2α and CRF2β, although CRF2β is also associated with the Golgi compartment.

CRF2α, but not CRF2β, has a non-cleavable pseudo signal peptide in the N-terminal that allows the formation of a protein complex with calnexin, an integral protein of the endoplasmic reticulum (Rutz et al., 2006; Schulz et al., 2010). Thus, it is possible that the difference in the N-terminals between both CRF2 isoforms could be responsible for the differences in their subcellular localization.

Different experimental approaches support that CRF2β forms a protein complex with D1R, like CRF2α (Fuenzalida et al., 2014). First, co-immunoprecipitation assays of recombinant proteins showed that CRF2β and D1R are in the same immunocomplex. Second, the use of the heteromer mobilization assay described by O’Dowd et al. (2005), allowed us to show that CRF2β follows the cellular distribution of D1Rnls, indicative of a stable protein complex. The stability of the protein complex is relevant for cellular functions such as signaling and endocytosis, among others (Tohgo et al., 2003).

The interaction between GPCRs is not required for ligand recognition or signaling, but may affect receptor mobilization and/or intracellular trafficking (Lohse, 2010). The formation of a heteromeric complex could change some of the GPCR properties such as the ligand affinity, intracellular signaling, desensitization, and recycling properties of the single receptors that compose the heteromeric complex. Several examples of these changes have been published (Juhasz et al., 2008; Magalhaes et al., 2010; Navarro et al., 2015). The presence of GPCRs mainly in intracellular compartments has been described before (Magalhaes et al., 2010; Chow et al., 2012). This subcellular localization of GPCRs is relevant for the assembly of a protein complex between them (Margeta-Mitrovic et al., 2000; Magalhaes et al., 2010). Further studies should evaluate how the difference in the subcellular localization between CRF2α and CRF2β impacts in their heteromerization with D1R, and how the presence of ligands influences in their assembly or disassembly with D1R (O’Dowd et al., 2011). The high identity in amino acid sequence that share both CRF2 receptors allow suggesting that a common domain could be responsible for the interaction of CRF2 with D1R.

The functional implications of the interaction between CRF2β and D1R could involve modulation of their actions in brain regions such as the septum and hippocampus, where both receptors are co-expressed and various peripheral areas such as the renal system and/or the blood circulatory system (Jose et al., 1998; Takahashi, 2012). Interestingly, the expression of both CRF2β and D1R is well documented in the kidney and the heart (Seeman and Grigoriadis, 1987; Yamaguchi et al., 1993; Ozono et al., 1997; Tezval et al., 2009; Takahashi, 2012). Dopamine controls ion transport and inflammatory response in the kidney (Armando et al., 2011) and even though the role of CRF2β is still not understood, it has been proposed that it could be implicated in vascular relaxation on renal arteries (Sanz et al., 2003). It has not been shown the presence of the D1R/CRF2beta complex in human cells. However, the presence of both receptors in the same cells in human kidney and heart tissue gives anatomical support for their eventual heteromerization.

In summary, the formation of a CRF2β and D1R protein complex may be therefore a potential therapeutic target for some brain disorders as well as cardiovascular or renal diseases. Thus, our data provide a new molecular target with new potential pharmacological properties.

## Data Availability Statement

The datasets generated for this study are available on request to the corresponding author.

## Author Contributions

HY, MA, and KG designed the experiments. HY performed the experiments and wrote the manuscript. HY, MA, and KG edited the manuscript.

## Funding

This work was funded by FONDECYT grants No 1150244 and 1191274 from CONICYT. HY was recipient of a doctoral fellowship from CONICYT.

## Conflict of Interest

The authors declare that the research was conducted in the absence of any commercial or financial relationships that could be construed as a potential conflict of interest.

The handling editor declared a past co-authorship with one of the authors [MA].
